# Prognostic significance of metabolic enzyme pyruvate kinase M2 in breast cancer: A meta-analysis

**DOI:** 10.1097/MD.0000000000008690

**Published:** 2017-11-17

**Authors:** Yiming Yang, Ke Wu, Yulin Liu, Liang Shi, Kaixiong Tao, Guobin Wang

**Affiliations:** a Department of Gastrointestinal Surgery; b Laboratory of Laparoscopic Surgery, Union Hospital, Tongji Medical College, Huazhong University of Science and Technology, Wuhan, China.

**Keywords:** breast cancer, overall survival, progression free survival, pyruvate kinase M2

## Abstract

**Backgrounds:**

Numerous studies have reported that aberrant pyruvate kinase M2 isoform (PKM2) expressed in cancer, indicating that PKM2 plays a critical role in tumor initiation and progression. Nevertheless, its prognostic value in breast cancer tumor is yet contentious. Therefore, we performed this meta-analysis to evaluate the prognostic significance of PKM2 in breast cancer.

**Methods:**

Eligible relevant literatures were retrieved by searching PubMed, the Cochrane Library, Embase through December 2016. Articles that comparing different PKM2 expression levels in human breast cancer tissues and prognostic significance were included. Software RevMan 5.3 and STATA (Review Manager (RevMan): [Computer program]. Version 5.3. Copenhagen: The Nordic Cochrane Centre, The Cochrane Collaboration, 2014. STATA: StataCorp. 2011. Stata Statistical Software: Release 12. College Station, TX: StataCorp LP) were applied to analyze the outcomes. Pooled results were presented in hazardous ratios (HRs) of 5-year overall survival (OS), progression-free survival (PFS), and odds ratios (ORs) of clinicopathological features with 95% confidence intervals.

**Results:**

Data from 6 involved studies with 895 patients were summarized. Breast cancer patients with high PKM2 had a worse OS (pooled HR = 1.65, 95% CI = 1.31–2.08, *P* < .001) and PFS (pooled HR = 2.49, 95% CI = 1.84–3.36, *P* < .00001). High PKM2 expression is related to lymph node metastasis (N1+N2+N3 vs N0, OR = 1.97, 95%CI = 1.39–2.80, *P* = .0001). The outcome stability was verified via sensitivity analysis. But elevated PKM2 expression was not correlated to tumor stage (T2+T3 vs T1, pooled OR = 0.80, 95% CI = 0.36–1.77, *P* = .58) and differential grade (G2+G3 vs G1, OR = 2.74, 95%CI = 0.76–9.84, *P* = .12). No publication bias was found in the included studies for OS (Begg test, *P* = .260; Egger test, *P* = .747).

**Conclusions:**

High PKM2 expression denotes worse OS and PFS in breast cancer patients, and correlate with the lymph node metastasis. However, there is no evidence for the impact of PKM2 expression on T stage and tumor differentiation.

## 1 Introduction

Breast cancer is the most prevalent female malignancy and causes a major cancer mortality worldwidely.^[1]^ In 2012, according to GLOBALCAN estimates, there were 1,676,600 new cases and 521,900 patients’ dead of breast cancer all over the world.^[2]^ Currently, therapeutic approaches including surgery, chemotherapy, radiotherapy, hormonal therapy, and Molecular targeted therapy are applied in clinical practice and have achieved considerable effect. Nevertheless, the long-term survival outcomes remain unsatisfied among high-risk individuals.^[3]^ Therefore, new efficient prognostic markers are needed to be identified for risk estimation in breast cancer patients.

Numerous oncological studies focus on cancer metabolism due to the aberrant feature of energy production. Tumor cells acquire the vast majority of energy from glycolysis and lactic acid fermentation regardless of sufficient oxygen supply, this unique phenomenon is known as Warburg effect, or aerobic glycolysis.^[4]^ The increased glycolysis confers tumor cell a proliferation advantage by promptly transforming glucose into some intermediates and substrates such as carbon, adenosine triphosphate (ATP), acetyl-CoA, nicotinamide adenine dinucleotide phosphate (NADPH) for tumor growth, glycolysis provides cells with these intermediates and substrates in adequate ratio for the synthesis of nucleotide, proteins, and membrane components.^[5,6]^ Meanwhile, this metabolic reprogramming permits cancer cells to survive under hypoxic environment.^[7]^

Cancer cells always greatly upregulate or downregulate glycolytic enzymes for metabolic reprogramming. Pyruvate kinase (PK) is a rate-limiting glycolytic enzyme that converts phosphoenolpyruvate (PEP) and adenosine diphosphate, into pyruvate and ATP irreversibly thereby determines glycolytic activity.^[8]^ In tumor cells pyruvate kinase predominantly presents as M2 isoform (PKM2) while normal cells express the M1 isoform (PKM1).^[9]^ Some studies have demonstrated that PKM2 plays an important role in breast cancer. However, the clinical significance of PKM2 expression remains controversial because of conflicting clinical evidence. Hence, we performed this meta-analysis to clarify the prognostic significance of PKM2 in human breast cancer and offer referential information for future clinical practice.

## 2 Materials and methods

### 2.1 Literature search

Following the PRISMA guidelines, databases including PubMed, Web of Science, Embase (via Ovid), and Cochrane Library were systematically examined use the search terms: “PKM2 OR pyruvate kinase isoform M2 OR pyruvate kinase isozyme M2 OR pyruvate kinase M2” with “breast cancer.” Each of the abstract and full-text of preliminary entries has been screened to guarantee the eligibility of involved studies.

### 2.2 Selection criteria

Studies that conform to the following criteria were ultimately included: formally published and with full-text English-written articles until December 2016; defined the PKM2 expression level; compared the prognostic value of different level of PKM2 expression in human breast cancer; provided adequate data to calculate the hazardous ratio (HR) of the effective index.

Studies were eliminated due to the following reasons: article type as reviews, comments, letters, case-reports; duplicate publication or data overlap; insufficient survival data for analysis;

### 2.3 Data extraction

Relevant data among the included documents were independently extracted from texts, tables, and figures by 2 professional reviewers. Any inconsistence in data extraction was fixed through cross check and discussion. The baseline characteristics was collected: first author, published year, country, patient volume, age distribution, detection method, cut off value of PKM2 expression, clinicopathological features (TNM stage), and survival data (5-year overall survival/progression free survival). Engauge Digitizer 4.1 (free software downloaded from http://sourceforge.net). was used to extract the survival data from some publications that only provided OS/PFS data in Kaplan–Meier curves.

### 2.4 Statistical analysis

The software Review Manager 5.3 (free software downloaded from http://www.cochrane.org) was employed to integrate data. The effect size was presented by HR and the corresponding 95% confidence interval (CI). A pooled HR >1 suggested a poor prognosis of patients with PKM2 positive or high-expression, on the contrary, HR <1 entailed a better one. *I*^2^ and *Q*-test indicated the degree of inconsistency across the included trails, *I*^2^ > 50% and *P* < .05 indicated uncompromising heterogeneity. Fixed-effects model or random-effects model was chosen for the low or high heterogeneity analysis, respectively. Sensitivity analysis was conducted via excluding low-quality studies and interchanging random effect model and fixed effect model among included trials to ensure the stability of pooled data. Moreover, Egger weighted regression test^[10]^ and Begg rank correlation test^[11]^ were applied to scrutinize publication bias amongst included studies. *P*-value <.05 implied statistical significance. All analyses adopted in this article were totally based on previous published studies, therefore, no ethical approval and consent from patients are required.

## 3 Results

### 3.1 Study selection process

**Figure 1:**
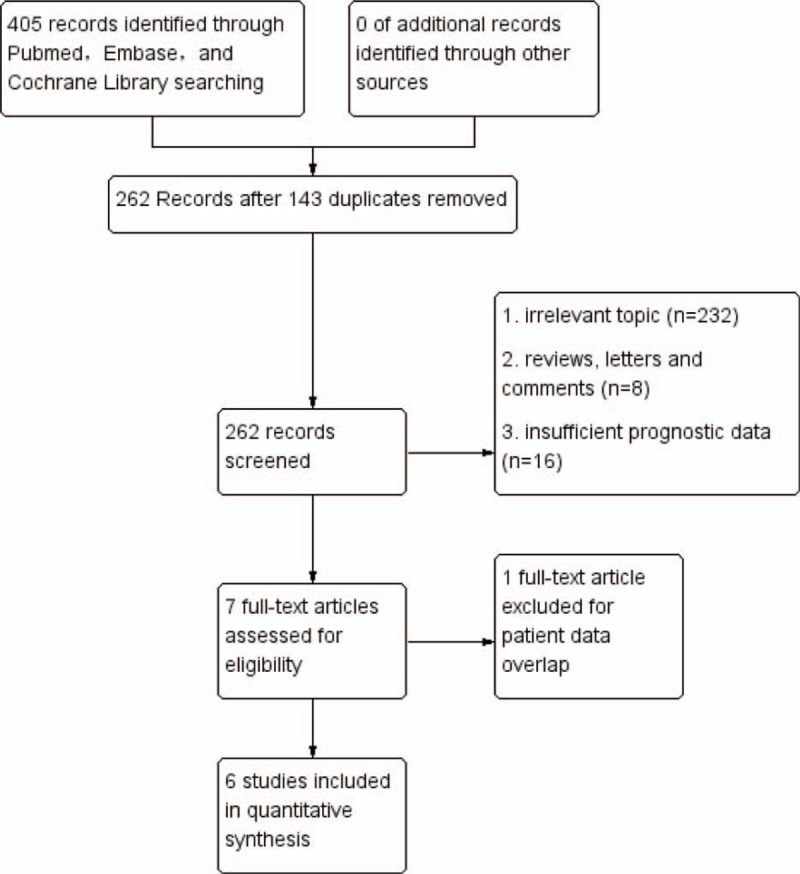
Study flow diagram for this meta-analysis.

The flowchart of Fig. 1 shows the literature search result. We primarily searched 405 studies from the databases described above. After excluding 143 duplicates, 262 full-text publications were left over to evaluate the eligibility. Next, 232 papers were excluded for irrelevant topic and 8 for inappropriate publication types, 16 for insufficient prognostic data. One of the left 7 studies was removed because its patient data overlapped with another left literature.^[12]^ Finally, 6 articles with 895 patients were included in the analysis.^[13–18]^

### 3.2 Study characteristics

Table 1 summarized the results of all included 6 studies. The sample volume of each study ranged from 20 to 296 with a total of 895 patients. The publication year extended from 2010 to 2016, 5 literatures originated from China and 1 from Germany. Six studies reported the 5-year OS and 3 studies describe the 5-year PFS. All the survival data were analyzed via Kaplan–Meyer method. HRs were directly extracted from the texts of 4 studies and survival curves of 3 studies. Four studies presented the correlation between PKM2 expression and clinicopathological information.

**Table 1:**
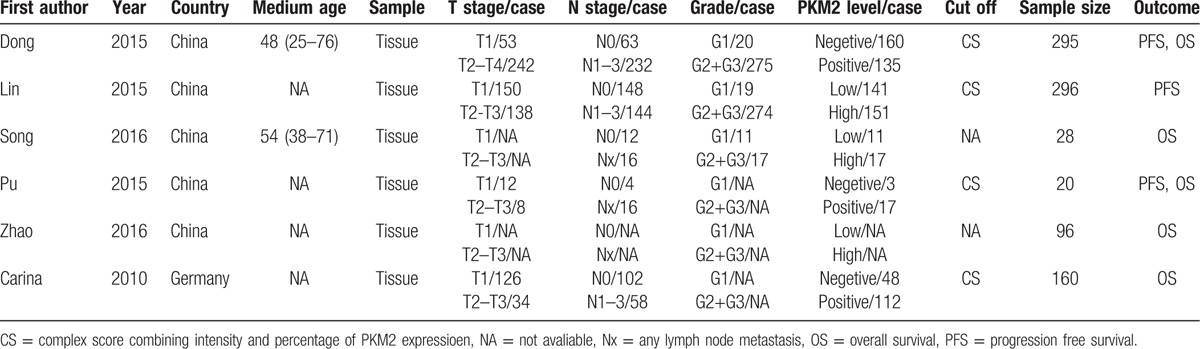
Main characteristics of included studies.

### 3.3 PKM2 expression and 5-year OS

**Figure 2:**
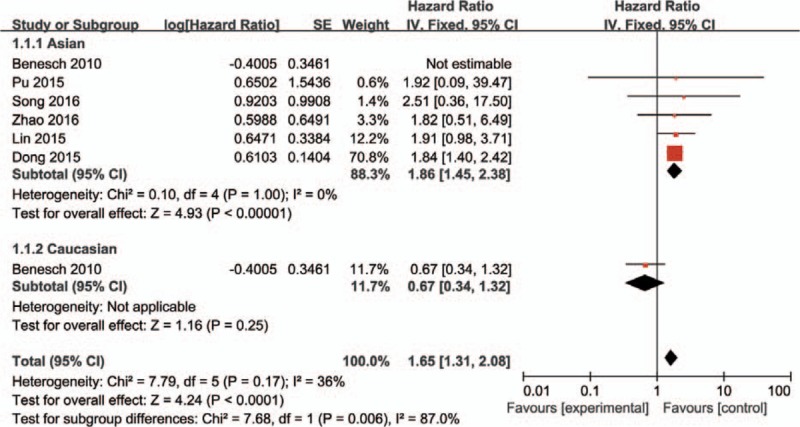
PKM2 expression and the 5-year OS. Six included studies investigated the correlation between PKM2 expression and OS. High PKM2 expression was highly correlated with a worse OS (HR = 1.65, 95%CI = 1.31–2.08, *P* < .001, fixed effect model). A subgroup analysis for ethnicity was performed, PKM2 is not a negative prognostic factor in Caucasians (HR = 0.67, 95%CI: 0.34–1.32, *P* = .25), but still a negative prognostic factor in Asians (HR = 1.85, 95%CI = 1.31–2.08, *P* < .00001). CI = confidence interval, HR = hazardous ratio, PKM2 = pyruvate kinase M2 isoform.

The combined analysis of 6 studies presented that high PKM2 expression was related to worse OS (pooled HR = 1.65, 95% CI = 1.31–2.08, *P* < .001) (Fig. 2). Due to the slight heterogeneity among included studies (*I*^*2*^ = 36%, *P* = .17), a fixed effect model was performed to pool HRs.

A subgroup analysis was performed to clarify the impact of ethnic factor. The result showed that PKM2 is not a negative prognostic factor in Caucasians (HR = 0.67, 95% CI = 0.34–1.32, *P* = .25), but still a negative prognostic factor in Asians (HR = 1.86, 95% CI = 1.45–2.38, *P* < .00001) (Fig. 2)

### 3.4 PKM2 expression and 5-year PFS

**Figure 3:**
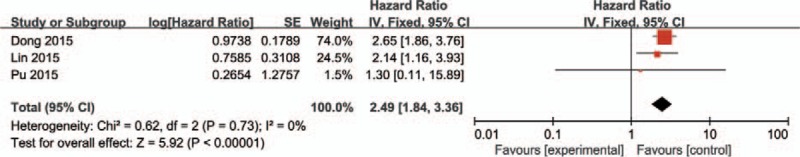
PKM2 expression and 5-year PFS. Three included studies investigated the correlation between PKM2 expression and PFS. High PKM2 expression was highly correlated with a poor PFS (HR = 2.49, 95%CI = 1.84–3.36, *P* < .0001, fixed effect model). CI = confidence interval, HR = hazardous ratio, PFS = progression-free survival, PKM2 = pyruvate kinase M2 isoform.

Three qualified studies were implemented to pool HRs for PFS via a fixed effect model since there was scarcely a statistical heterogeneity (*I*^*2*^ = 0.0%, *P* = .73). The results showed that elevated PKM2 expression denoted a negative clinical outcome in patients with breast cancer (pooled HR = 2.49, 95% CI = 1.84–3.36, *P* < .00001) (Fig. 3).

### 3.5 Correlation between PKM2 and clinicopathological features

**Figure 4:**
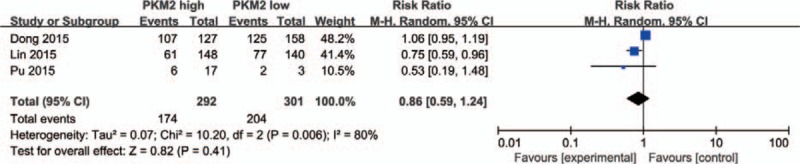
PKM2 expression and T stage. Elevated PKM2 expression was not correlated to tumor stage (T2+T3 vs T1, OR = 0.80, 95%CI = 0.36–1.77, *P* = .58, random effect model). CI = confidence interval, OR = odds ratio, PKM2 = pyruvate kinase M2 isoform.

Extracted data from 3 included studies that reported the correlation of PKM2 expression and T stage demonstrated that elevated PKM2 expression was not correlated to tumor stage (T2+T3 vs T1, pooled OR = 0.80, 95% CI = 0.36–1.77, *P* = .58) (Fig. 4). Uncompromising heterogeneity (*I*^*2*^ = 66%, *P* = .05) led to the usage of a random effect model.

**Figure 5:**
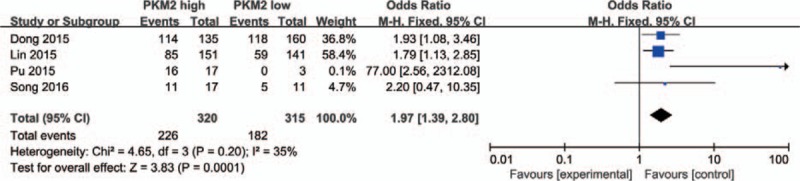
PKM2 expression and N stage. High PKM2 expression was related to lymph node metastasis (N1+N2+N3 vs N0, OR = 1.97, 95%CI = 1.39–2.80, *P* = .0001, fixed effect model). CI = confidence interval, OR = odds ratio, PKM2 = pyruvate kinase M2 isoform.

In addition, 4 studies provided the information between PKM2 expression and N stage. Pooled data indicated that high PKM2 expression is related to lymph node metastasis (N1+N2+N3 vs N0, OR = 1.97, 95%CI = 1.39–2.80, *P* = .0001) (Fig. 5), minor heterogeneity was found among the 4 studies (*I*^*2*^ = 35%, *P* = .20), therefore, a fixed effect model was adopted.

**Figure 6:**
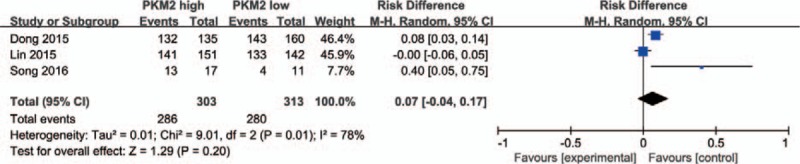
PKM2 expression and tumor differentiation. Differential grade was not correlated to PKM2 expression level (G2+G3 vs G1, OR = 2.74, 95%CI = 0.76–9.84, *P* = .12, random effect model). CI = confidence interval, OR = odds ratio, PKM2 = pyruvate kinase M2 isoform.

However, the differential grade was not correlated to PKM2 expression level (G2+G3 vs G1, OR = 2.74, 95%CI = 0.76–9.84, *P* = .12), random effect model (heterogeneity parameter *I*^2^ = 68%, *P* = .04). (Fig. 6)

### 3.6 Sensitivity analysis

In the first step, we excluding low-quality trials of Song and Zhao^[16]^ and Pu et al,^[17]^ results of OS (*P* < .0001), PFS (*P* < .00001), and N stage (*P* = .0009) confirmed the stability of our analysis.

Secondly, by changing the fixed-effects model to random-effects model in OS, PFS and N stage, outcomes of overall survival (HR = 1.51, 95%CI = 1.01–2.28 *P* = .05) and PFS (HR = 2.49, 95%CI = 1.84–3.36, *P* < .00001) and N stage (HR = 2.06, 95%CI = 1.21–3.52, *P* = .008) remain stable.

### 3.7 Publication bias

**Figure 7:**
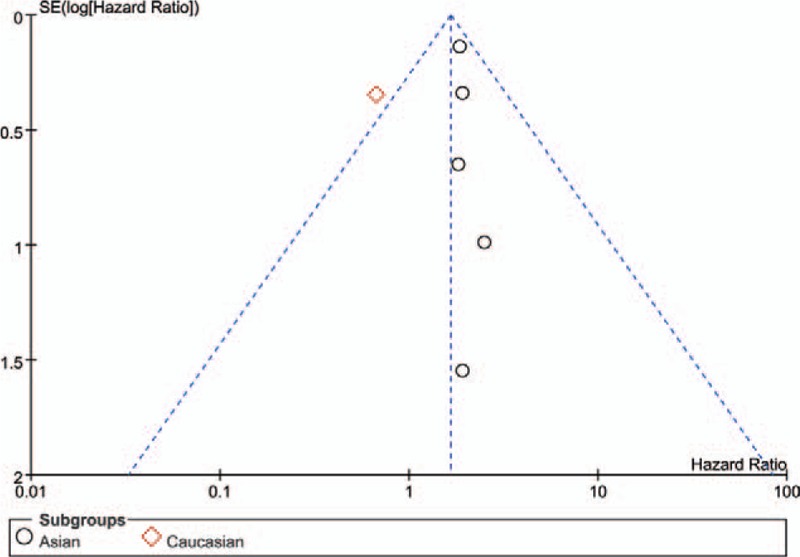
Funnel plots to evaluate publication bias of included studies for OS. OS = overall survival.

Funnel plots of included studies were drawn via RevMan 5.3. Take 5-year OS, for example, the almost symmetric result indicated that there was no evidence for a publication bias in this study (Fig. 6). Also, we performed Begg and Egger test in Stata software. (Begg test *P* = .260, Egger test *P* = .747) All the *P* values >.05 (Table 2) denoted the same conclusion as funnel plots suggested (Fig. 7).

**Table 2:**
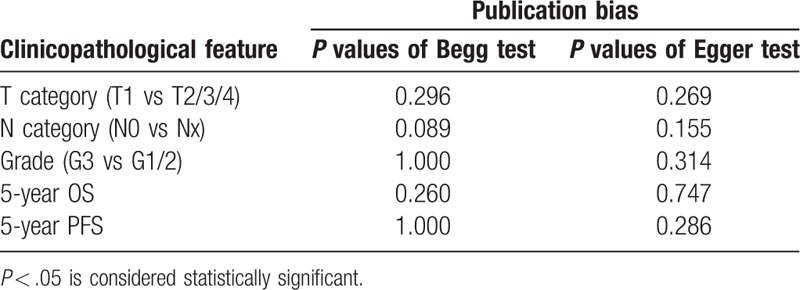
Egger test and Begg test for publication bias among included studies.

## 4 Discussion

The metabolic phenotypes that cancer cells possess, which are distinct from normal cells, play a critical role in cell development and proliferation. Necessary precursor elements for rapid cell proliferation were largely produced in the process of metabolic reprogramming of cancer cells.^[19,20]^ To be specific, cancer cells preferentially splice the primary RNA of PKM gene into PKM2 other than PKM1, which is a key enzyme in the final and rate-limiting reaction of glycolytic pathway, in order to promote aerobic glycolysis and, therefore, in favor of tumorigenesis.^[21]^ Moreover, expression of PKM2 confers extra advantages on cancer cells by balancing the concentration of intracellular reactive oxygen species (ROS) hence allowing them to withstand anti-oxidant responses in the environment of acute oxidative stress.^[22]^ In addition, PKM2 also presents non-metabolic functions via acting as a coactivator and protein kinase, which makes contribution to tumorigenesis.^[23]^ And increased cell adhesion mediated drug resistance was found to be correlated to PKM2 expression.^[24]^ Signal pathways that involve PKM2 including β-catenin, NF-κB, H3 T11, and c-Myc are basically related to tumorigenesis and proliferation.^[25]^

Despite many studies have described the correlation between PKM2 expression and the outcomes of cancer patients, the prognostic significance of PKM2 in breast cancer remains contentious. Benesch et al^[14]^ have reported that high PKM2 expression is related to positive outcomes in breast cancer. While Dong et al^[13]^ showed a poor prognosis in breast cancer with elevated PKM2 expression. We notice that a meta-analysis written by Zhu et al^[26]^ reveals the correlation between PKM2 and solid tumors including breast cancer. Regarding that Zhu et al only included 2 studies with 591 patients and conclude the overall survival and PFS in their manuscript, we investigated 6 screened individual studies from PubMed, Web of Science, Embase (via Ovid), and Cochrane Library databases with a sum of 895 cases to assess the clinical value including OS, 5-year PFS, T stage, lymph node metastasis, and tumor differentiation of PKM2 in subjects with breast cancer. According to our meta-analysis results, high expression of PKM2, which indicates a favor of tumor cell initiation and progression, is correlated to a poor prognosis due to worse 5-year overall survival and disease free survival in patients with breast cancer.

In breast cancer, targeting PKM2 seems to be a promising treatment. Li et al^[27]^ reported that PKM2 inhibitor shikonin enhanced the sensitivity of breast cancer cell to taxol and prolonged animal survival and reduced tumor size. Cyclosporine A was also an efficacious inhibitor of PKM2 and able to impair breast cancer cell proliferation.^[28]^ Considering the complex function of PKM2 in cell biology, measures that inhibiting or silencing PKM2 possibly cause a wide range of effects in human body. Therefore, the therapeutic value of PKM2 should be systematically assessed.

It is worth noting that among the 6 studies that provided OS data, 1 from Germany presents a contradictive conclusion to the other 5 Chinese articles. Thus, we performed a subgroup analysis to clarify the impact of ethnic factor. The result showed that PKM2 is not a negative prognostic factor in Caucasians. Lockney et al^[29]^ also found that elevated PKM2 expression is correlated to a positive outcome in Caucasians with pancreatic cancer. Possibly PKM2 expression denotes better prognosis in Caucasian population but a worse one in Asians.

In our study, a tendency of lymph node metastasis was found in high PKM2 expression breast cancer according to the pooled result of included 4 publications. Appropriate explanation may be related to vascular endothelial growth factor C (VEGF-C) which contributes to metastasis through improving tumor-initiating cell-associated characteristics.^[30]^ Hypoxia-induced factor-1 alpha (HIF1-α) could shift cell glycolytic reprogramming and upregulation of phosphoinositide dependent kinase(PDK)1–3 and PKM2.^[31]^ HIF1-α also upregulated VEGF-C which promotes lymphangiogenesis and angiogenesis in patients with breast cancer.^[32]^ In a word, PKM2 and VEGF-C are simultaneously upregulated in breast cancer by HIF1-α and VEGF-C plays a critical role in the mechanism of lymph node metastasis.

However, a few limitations in our study should be admitted. First, although we have not found any obvious evidences for publication bias from funnel plots, Egger and Begg tests, this meta-analysis was based on formally published articles with principally positive results. Hence, there is a potential publication bias that lowers the accuracy and validity of the results. Second, due to some relatively small sample studies and some missing information, the qualities of each included studies are not in the same level. Third, the inconsistences of cut-off values and experimental designs in the included studies may contribute to heterogeneity. Unfortunately, we failed to conduct subgroup analyses to discover these influences because of the insufficiency of detailed data. Fourth, some studies merely provided survival curves other than direct HR values, that may result in some slight deviations from the authentic HRs.^[33]^ Finally, the heterogeneity of the clinical features of the patients cannot be ignored, especially the Asian ethnicity occupied the vast majority of included studies, thus the pooled outcome in Caucasians might not be convincing owing to the lack of enough sample.

In conclusion, this meta-analysis reveals that high PKM2 expression denotes worse OS and PFS in breast cancer patients, and correlate with the lymph node metastasis. However, there is no evidence for the impact of PKM2 expression on T stage and tumor differentiation. PKM2 might be a potential prognostic biomarker and therapeutic target for breast cancer.

## Acknowledgment

The authors thank their colleagues in their laboratory for methodological support.
